# Occurrence of Impostor Syndrome in Dentistry Faculty and Related Factors

**DOI:** 10.1111/eje.70032

**Published:** 2025-08-21

**Authors:** Matheus Lima Silva da Rocha, Marcos Diego Lima de Oliveira, Amanda de Carvalho Taveira Gomes, Karolyne de Melo Soares, Basílio Rodrigues Vieira, José Washington de Morais Medeiros, José Maria Chagas Viana Filho

**Affiliations:** ^1^ Centro Universitário UNIESP Cabedelo PB Brazil; ^2^ Universidade Federal da Paraíba João Pessoa PB Brazil; ^3^ Universidade Estadual da Paraíba Campina Grande PB Brazil; ^4^ Instituto Federal da Paraíba João Pessoa PB Brazil; ^5^ Universidade de Pernambuco Arcoverde PE Brazil

**Keywords:** dentistry, impostor phenomenon, impostor syndrome, teaching

## Abstract

**Aim:**

To investigate the occurrence of Impostor Syndrome (IS) among faculty members of undergraduate dental courses at institutions in a capital city in the Northeast of Brazil.

**Materials and Methods:**

A quantitative cross‐sectional observational study was conducted involving five higher education institutions. Data collection was performed through a structured online questionnaire composed of two domains: sociodemographic data and the Clance Impostor Phenomenon Scale (CIPS). Responses were analysed using descriptive and inferential statistics to identify differences and associations related to gender, type of institution and teaching experience (in years). A significance level of *p* ≤ 0.05 was adopted.

**Results:**

The study included 65 professors with a median age of 37 years (*p* ^25^ = 32; *p* ^75^ = 46), predominantly female (72.3%, *n* = 47), teaching in private institutions (73.8%, *n* = 48) and with a median year of teaching experience of 7 years (*p* ^25^ = 4; *p* ^75^ = 13). The median IS score was 42 (*p* ^25^ = 35; *p* ^75^ =54), indicating a moderate level. A difference in IS scores was observed between professors from public and private institutions (*p* = 0.049; *r* = 0.322; 95% CI: 0.085 to 0.525) and a negative correlation between IS and years of teaching experience as continuous variables (*p* = 0.006; *r* = −0.335; CI = −0.099 to −0.535), suggesting that IS tends to decrease with increased years of teaching experience.

**Conclusion:**

Moderate levels of IS were observed among dental faculty members teaching undergraduate courses. The findings suggest that professors working in private institutions and those with fewer years of teaching experience may be more susceptible to IS.

## 1 Introduction

Impostor Syndrome (IS) is a psychological phenomenon in which individuals, even in the face of personal achievements and competencies, attribute their success to external factors such as luck or chance, perceiving themselves as ‘frauds’ in the eyes of others [1, 2, 3, 4]. Initially described by Clance and Imes (1978) [1] in successful women, subsequent studies have demonstrated that IS also affects men [5, 6] and is associated with an overemphasis on personal shortcomings, rejection of praise and a persistent fear of exposure [2, 7, 8].

In the occupational context, IS is linked to stress [9, 10, 11] and Burnout Syndrome (BS), which undermine professional well‐being and quality of life [12, 13]. Its aetiology involves multiple factors, including perfectionism, anxiety, low self‐esteem, family background and belonging to social minorities [7, 14, 15, 16]. In academia, IS is driven by high demands, competitiveness and institutional pressure; it is frequently reported among university faculty who face elevated expectations for performance and productivity [10, 17, 18, 19].

The Clance Impostor Phenomenon Scale (CIPS), developed in 1985, is widely used to identify and measure IS in different contexts [20, 21], including higher education [17, 22, 23, 24, 25]. Studies utilising the CIPS have shown a significant prevalence of this phenomenon in academic environments, particularly in the health sciences [7, 17, 22, 24].

Investigating IS among dental professors is particularly relevant, as these professionals operate in a high‐demand environment that requires ongoing education, scientific output, clinical skills and teaching expertise [25, 26]. The pressure to achieve results, academic competitiveness [27] and the pursuit of excellence in dental education and patient care can increase the susceptibility of these faculty members to the impostor phenomenon, affecting both their mental health and the quality of education provided [24, 27].

To date, this is the first study to investigate the occurrence and associated factors of Impostor Syndrome among faculty members of undergraduate dental programmes. Thus, the aim was to analyse the prevalence of IS in this population of faculty members from public and private institutions in a capital city in Northeastern Brazil, based on the hypothesis that factors such as gender, type of institution and years of teaching experience influence the levels of IS among dental faculty.

## 2 Materials and Methods

### 2.1 Study Design and Ethical Considerations

This cross‐sectional observational study was conducted in accordance with the STROBE (Strengthening the Reporting of Observational Studies in Epidemiology) guidelines. The research was approved by the Research Ethics Committee of the UNIESP University Center (Opinion number: 5.249608 / CAAE: 55900922.8.0000.5184). All participants provided electronic informed consent prior to participation.

### 2.2 Study Setting and Sample Selection

The study was conducted in João Pessoa, Paraíba, Brazil, and involved all five higher education institutions offering undergraduate dental programmes at the time of data collection (July 2022 to July 2023). The total population of faculty members was obtained from official institutional records, totalling 201 individuals.

The estimated sample size of 61 participants was calculated prior to data collection using the OpenEpi v.3.01 tool, considering the population of 201 faculty members, regardless of their specific curricular assignment, provided they held a degree in Dentistry. The calculation did not stratify by type of institution (public or private). The response rate was calculated by dividing the number of completed questionnaires received by the total number of eligible faculty members invited to participate.

Faculty members were recruited via WhatsApp and through survey links distributed by course coordinators. Inclusion criteria comprised contracted faculty from private institutions and permanent or substitute professors from public institutions who taught undergraduate dentistry courses in João Pessoa and completed the questionnaires fully and correctly. Professors responsible for basic curricular courses were included if they held a dental degree. Exclusion criteria included professors from other regions, professors linked only to postgraduate programmes, non‐dentist undergraduate professors, and those who requested removal of their responses.

Exclusion criteria were applied prior to data collection. No replacement of participants was performed in cases of incomplete or duplicate responses. In cases of non‐response or incomplete questionnaires, these questionnaires were excluded from the analysis.

### 2.3 Data Collection and Analysis

Data were collected between July 2022 and July 2023 using a structured online questionnaire (Google Forms). The researcher initially contacted the course coordinators at the five institutions via WhatsApp and, upon positive feedback, presented the survey for distribution to their respective faculty groups.

Before responding, participants were informed about the objectives of the research through the Informed Consent Form (ICF). Upon agreement, participants accessed the data collection instrument, which was divided into two domains: Domain 1—sociodemographic questions (gender, age, length of teaching experience); Domain 2—Clance Impostor Phenomenon Scale (CIPS). The variable ‘gender’ was collected according to participants' self‐identification and was analysed in a standardised manner.

The CIPS, originally developed by Clance (1985) and adapted for the Brazilian context by Bezerra et al. (2021), consists of 20 items rated on a 5‐point scale, ranging from 1 (Does not describe me) to 5 (Describes me completely). Higher scores indicate a greater presence of impostor traits; scores were classified as follows: low (≤ 40), moderate (41–60), high (61–80) and very high (> 80).

To ensure data integrity, participants were instructed to respond only once, and email addresses were collected to identify and remove duplicate entries. All responses were exported from Google Forms to Microsoft Excel, where consistency checks were performed. Incomplete questionnaires and duplicates were excluded from the analysis. No imputation was performed for missing data; only fully completed responses were included.

The data obtained were analysed using descriptive and inferential statistics with Jamovi software (version 2.3.12). The normality of numerical data was checked using the Shapiro–Wilk test (*p* < 0.05). The Mann–Whitney U‐test was used to compare IS scores across gender, type of institution (public/private) and length of years of teaching experience (≤ 10 years vs. > 10 years). Fisher's exact test was used for associations between answers to each item on the CIPS and the same variables. Pearson's correlation test was used to analyse the relationship between IS scores and years of teaching experience. For all tests, *p* ≤ 0.05 was considered significant.

In addition to bivariate analyses, a multinomial logistic regression model was performed to evaluate the independent effects of gender, type of institution and years of teaching experience on IS levels, controlling for potential confounders.

## 3 Results

The sample consisted of 65 faculty members (response rate = 32.3%), with a median age of 37 years (*p* ^25^ = 32; *p* ^75^ = 46); the majority of whom were female (*n* = 47; 72.3%), who taught at private institutions (*n* = 48, 73.8%) and had a median of 7 years of teaching experience (*p* ^25^ = 4; *p* ^75^ = 13). The median score IS among undergraduate dental professors was 42 (*p* ^25^ = 35; *p* ^75^ = 54), corresponding to a moderate level of the syndrome, as shown in Table 1.

**TABLE 1:** Sociodemographic and professional characteristics of undergraduate dentistry faculty members in João Pessoa, Paraíba, Brazil (*n* = 65).

Characterisation of the sample
Age (in years)
37 (*p* ^25^ = 32.0; *p* ^75^ = 46.0)
Gender
Male–18 (27.7%) Female–47 (72.3%)
Type of institution you work for
Public–17 (26.2%) Private–48 (73.8%)
Teaching time (in years)
7 (*p* ^25^ = 4.0; *p* ^75^ = 13.0)
Level of Impostor syndrome
42 (*p* ^25^ = 35.0; *p* ^75^ = 54.0)

*Note:* Numerical variables: median (percentile^25^; percentile^75^); nominal variables: *n* (%).

*Source:* Authorship, 2025.

Table 2 presents the comparison of Impostor Syndrome (IS) scores according to gender, type of institution and years of teaching experience, considering IS as a discrete quantitative variable. The results indicated a tendency towards higher IS scores among professors from private institutions compared to those from public institutions (*p* = 0.049; *r* = 0.322; 95% CI: 0.085–0.525). No significant differences were identified between genders (*p* = 0.407) or between the years of teaching experience groups (≤ 10 years and > 10 years; *p* = 0.082).

**TABLE 2:** Comparison of impostor syndrome (IS) scores according to gender, type of institution and years of teaching experience among undergraduate dentistry faculty in João Pessoa, Paraíba, Brazil *n* = 65).

Variable	IS – MED (*p* ^25^; *p* ^75^)	%	*p*	*r*	95% IC
Gender					
Male Female	41 (*p* ^25^ = 33.5; *p* ^75^ = 48.0) 42 (*p* ^25^ = 35.5; *p* ^75^ = 57.0)	27.7 72.3	0.407	0.134	−0.114 – 0.365
Type of institution you work for					
Public Private	38 (*p* ^25^ = 30.0; *p* ^75^ = 48.0) 44 (*p* ^25^ = 38.0; *p* ^75^ = 55.0)	26.2 73.8	0.049*	0.322	0.085–0.525
Teaching time					
≤ 10 years > 10 years	43 (*p* ^25^ = 38.0; *p* ^75^ = 57.3) 38 (*p* ^25^ = 28.0; *p* ^75^ = 50.0)	64.6 35.4	0.082	0.262	0.020–0.477

*Note:* *p*: < 0.05*; *p* ^25^: percentile^25^; *p* ^75^: percentile^75^; *r*: effect size. *Source:* Authorship, 2025.

Abbreviations: %, percentage frequency; 95% CI, confidence intervals; IS, Impostor Syndrome; Mann–Whitney U‐test; MED, median.

To determine whether the associations identified in the bivariate analyses would persist after adjusting for potential confounding variables, a multinomial logistic regression was performed. The model included institution type (public/private), years of teaching experience (> 10 years/ ≤ 10 years) and gender (female/male) as predictors.

The results showed that only institution type remained statistically significant for the moderate level of IS compared to the low level. Faculty members from private institutions had 4.65 times higher odds of reporting a moderate level of IS compared to those from public institutions (OR = 4.65; 95% CI: 1.09–19.86; *p* = 0.038). The other predictors and outcome levels did not show statistically significant associations in the multivariable model (Table 3). The goodness of fit of the multinomial logistic regression model was assessed using Deviance, AIC and Cox & Snell pseudo‐R^2^ values, which indicated an acceptable fit for the data (Deviance = 134; AIC = 158; R^2^CS = 0.0730–0.0931).

**TABLE 3:** Multinomial logistic regression analysis of factors associated with different levels of impostor syndrome (IS) among undergraduate dental faculty (*n* = 65).

Impostor syndrome level (vs. low)	Predictor	OR	95% CI	*p*
Moderate	Private Institution	4.645	1.09–19.86	0.038*
	> 10 years teaching	0.675	0.20–2.33	0.534
	Female	1.081	0.31–3.81	0.903
High	Private Institution	2.329	0.39–14.03	0.356
	> 10 years teaching	0.809	0.15–4.23	0.802
	Female	1.511	0.25–9.15	0.654
Very high	Private Institution	Not estimated	Not estimated	Not estimated
	> 10 years teaching	Not estimated	Not estimated	Not estimated
	Female	Not estimated	Not estimated	Not estimated

*Note:* *: *p* < 0.05. *Source:* Authorship, 2025.

Abbreviations: 95% CI, confidence intervals; OR, Odds ratios; Reference category, Low IS.

The faculty members' responses to each item on the Scale Clance of the Impostor Phenomenon were analysed to check for possible associations with the variables gender, type of institution and years of teaching experience. The results are shown in Table 4. For interpretation, note that for the question ‘(1) I have often succeeded in a test or task even though I was afraid I wouldn't do well before I understood it’, the response ‘Doesn't describe me’ (answer 1) was associated with professors from public institution (*p* = 0.035), while the response ‘Describes me completely’ (answer 5) was associated with having more than 10 years of teaching experience (*p* = 0.036). In addition, in the question ‘(2) I can give the impression that I am more competent than I really am’, there was an association between faculty members with 10 or more years' experience and the answer ‘Does not describe me’ (answer 1). On the other hand, those with less than 10 years of teaching experience were associated with the answer ‘Rarely describes me’ (answer 2) (*p* = 0.036).

**TABLE 4:** Associations between gender, type of institution, teaching experience and individual responses to the Clance Impostor Phenomenon Scale among undergraduate dentistry faculty in João Pessoa, Paraíba, Brazil (*n* = 65).

Questions	Gender		*p*	Type of institution		*p*	Teaching time		*p*
	Male (*n* = 18)	Female (*n* = 47)		Public (*n* = 17)	Private (*n* = 48)		< 10 Years (*n* = 42)	≥ 10 Years (*n* = 23)	
1) I've often succeeded in a test or task even though I was afraid I wouldn't do well before I understood it	—	—	—	Answer 1 (*n* = 02)	—	**0,035** *	—	Answer 5 (*n* = 04)	**0,036** *
2) I can give the impression that I'm more competent than I really am	—	—	—	—	—	—	Answer 2 (*n* = 12)	Answer 1 (*n* = 11)	**0,036** *
7) I tend to remember situations in which I didn't do my best more than situations in which I did my best	—	—	—	Answer 1 (*n* = 10)	Answer 3 (*n* = 14)	**0,033** *	—	—	—
13) Sometimes I'm afraid that others will find out how much knowledge or skill I really lack	—	—	—	—	—	—	Answer 3 (*n* = 12)	Answer 1 (*n* = 16)	**0,029** *
18) Sometimes I worry that I won't do well on a project or in an exam, even though other people close to me are confident that I'll do well	—	—	—	—	—	—	Answer 3 (*n* = 18)	Answer 5 (*n* = 04)	**0,010** *
19) If I'm about to receive a promotion at work or be recognised for something, I hesitate to tell other people until it actually happens	Answer 3 (*n* = 09)	Answer 4 (*n* = 15)	**0,018** *	—	—	—	—	—	—

*Note:* Answers: (1) Doesn't describe me, (2) Rarely describes me, (3) Describes me occasionally, (4) Describes me often, (5) Describes me completely. *Source:* Author, 2025. Bold values indicate Fisher's exact test (*p* < 0.05).

* Fisher's exact test (*p* < 0.05); values adjusted using the Bonferroni method; *n*: absolute frequency; *p*: significance.

**FIGURE 1:**
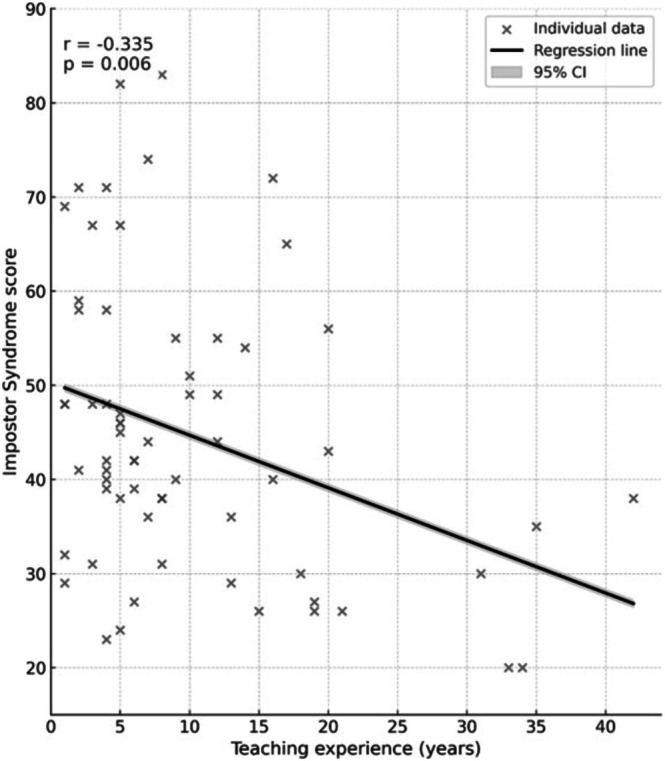
Correlation between years of teaching experience (in years) and Impostor Syndrome (IS) scores among undergraduate dentistry faculty members in João Pessoa, Paraíba, Brazil (*n* = 65). *Source:* Authorship, 2025.

The correlation test between the quantitative variables discrete, IS and teaching time in years, revealed a negative correlation significant (*r* = −0.335; IC: −0.099 – −0.535; *p* = 0.006), as illustrated in Figure 1. This indicates that IS tends to decrease as teaching time increases.

## 4 Discussion

This is the first study to investigate the prevalence and associated factors of Impostor Syndrome (IS) among undergraduate dentistry faculty in Brazil, identifying a predominance of moderate syndrome levels in this population. These findings are particularly relevant given the professional and psychological repercussions of IS, including excessive fear of failure, work overload and the potential development of conditions such as BS [12, 13].

Impostor syndrome can impair both professional performance and the quality of life of dental faculty, being associated with self‐sabotage and persistent feelings of inadequacy [17]. Recent studies have indicated that IS may be associated with severe outcomes, including suicidal ideation among medical students [27], and tends to be intensified in competitive and high‐risk academic environments such as health courses [17]. These data reinforce both the need and call [28] for institutions to value not only academic excellence but also emotional and psychological support.

In the present study, a trend towards higher IS scores was observed among faculty from private institutions compared to those from public institutions, a result that was confirmed in multivariate analysis, where teaching at a private institution was independently associated with moderate IS levels (OR = 4.65; 95% CI: 1.09–19.86; *p* = 0.038). It is important to note, however, that this association was not observed for high or very high IS levels, and the confidence interval for the OR was relatively wide, indicating that these findings should be interpreted with caution. Moreover, the comparisons between public and private institutions were exploratory and secondary, as the sample size was calculated for the overall population and not stratified by institution type, which may have limited the statistical power of these analyses. As such, these results should primarily serve to guide and inform future research with larger, stratified samples.

Previous studies suggest that academic environments marked by contractual instability, greater performance demands and less institutional support—characteristics often found in private institutions—may increase insecurity and vulnerability to the impostor phenomenon, especially among early‐career faculty [2, 19, 23]. However, IS is a multifactorial phenomenon, and factors not assessed in this study, such as organisational climate, peer support and personal coping strategies, may have contributed to the observed associations [3].

A negative correlation between years of teaching experience and IS scores was also identified (*r* = −0.335), indicating that more experienced faculty tend to report lower levels of impostor feelings. Similar results have been found in the literature, which point to accumulated experience as a protective factor, promoting self‐confidence, self‐efficacy and professional resilience [3, 23]. Early‐career faculty may be particularly susceptible to impostorism due to uncertainties regarding their professional roles, the need to build a reputation and frequent exposure to evaluation. However, the magnitude of the observed correlation was weak, suggesting that factors beyond years of teaching experience play an important role in IS.

No significant differences in IS scores were observed between male and female faculty, which is consistent with previous findings in higher education settings [3, 4, 7, 25]. This result may reflect social advances, greater gender equity in academia and female empowerment. However, qualitative studies highlight that men and women may adopt different coping mechanisms for IS: women often seek social support, while men tend to internalise their concerns or resort to compensatory work [14, 25]. These nuances reinforce the importance of mixed methodological approaches in future studies.

Additionally, the analysis of individual responses from the Clance Impostor Phenomenon Scale revealed that specific aspects of the phenomenon—such as fear of evaluation and hesitancy to share achievements—vary according to years of teaching experience and type of institution. Such granularity points to the heterogeneity of IS and the need for multifaceted assessment and intervention strategies.

Regarding faculty development, postgraduate education plays a fundamental role in the training of faculty members and researchers, providing advanced scientific knowledge, investigative skills and pedagogical abilities [28, 29]. However, obtaining a master's or doctoral degree represents only one stage of professional development.

It is essential for faculty to remain engaged in ongoing professional development activities, such as educational workshops, mentoring programmes, participation in scientific conferences and integration into academic communities of practice. Such actions are essential for maintaining teaching quality, career satisfaction and the prevention of IS over time [29]. Institutional policies that promote continuous training, career planning, mentorship and psychosocial support contribute significantly to faculty well‐being and professional longevity [29, 30].

This study presents several limitations that should be considered. The sample size was modest, and there was an imbalance between the number of faculty members from public and private institutions, which may have limited the statistical power and the generalisability of between‐group comparisons. In addition, the response rate was 32.3%, resulting in a relatively high non‐response rate (67.7%). This phenomenon has been reported in prior research on academic professionals [31], particularly when self‐administered questionnaires are used, largely due to participants' workload and limited availability [32]. Nevertheless, it is important to emphasise that the number of respondents exceeded the minimum sample size previously calculated, thereby ensuring the robustness of the statistical analyses performed.

Furthermore, although some potential confounders were included in the multivariate analysis, other relevant factors—such as organisational climate, professional autonomy and job satisfaction—were not assessed and may have influenced the findings. Given the exploratory and cross‐sectional design of this study, the results should be interpreted with caution. Therefore, it is recommended that future research use larger and more representative samples, adopt longitudinal approaches and consider psychosocial and institutional variables in order to deepen the understanding of IS among dental faculty.

Despite these limitations, the findings reinforce the need for greater institutional recognition of IS and the implementation of comprehensive support strategies for dental faculty. Interventions such as mentoring programmes, access to psychological counselling, structured career planning and the promotion of an academic culture open to professional challenges may help mitigate the negative consequences of IS and foster healthier, more resilient and collaborative academic environments [23, 29, 30].

## 5 Conclusion

Moderate levels of IS were observed among dental faculty members teaching undergraduate courses. The findings suggest that professors working in private institutions and those with fewer years of teaching experience may be more susceptible to IS. Given the exploratory and cross‐sectional nature of this study, further research with larger and more diverse samples and more robust methodological designs is needed to confirm these associations and better understand the factors influencing IS among dental educators.

## Conflicts of Interest

The authors declare no conflicts of interest.

## Data Availability

The data that support the findings of this study are available on request from the corresponding author. The data are not publicly available due to privacy or ethical restrictions.
